# U. S. College of Veterinary Surgeons

**Published:** 1895-05

**Authors:** 


### U. S. COLLEGE OF VETERINARY SURGEONS.

The first annual commencement exercises of the United States College of Veterinary Surgeons was held in the college building, 222 C Street, N. W., Washington, D. C., on April 18th. The degree of the college was conferred upon Jacob K. Shaub, of Lancaster, Pa., and Mortimer R. Weiner, Buffalo, N. Y. The school had eleven matriculants at its opening in October, 1894. The graduating class was addressed by Dr. C. Barnwell Robinson, in which he gave strict injunctions as to the necessity of every graduate upholding the dignity of the profession and the integrity of the institution from which they were emerging.

The valedictory address was delivered by Prof. D. S. Lamb, who in well-chosen words and kindly advice bade the students Godspeed in their field of work.

The announcement was made that hereafter the school would adopt a two-years' course, providing the students who presented themselves for its degree are found thoroughly competent by the Board of Examiners who conduct the final examination.

A free scholarship was announced as being open to a student of the high school of the District of Columbia and to each agricultural college throughout the States.

---

The recent graduating class of the College of Pharmacy of Philadelphia found five young ladies among the list for the honor of her degree. Among the theses we note one entitled "Tasteless Fluid Extract of Cascara Sagrada," a remedy much used in canine practice, and which from its present bitter taste becomes very objectionable in administering, and every effort is made by these animals to discharge it. We hope this may become an article of commerce among drug dealers if it still retains its laxative properties.

Veterinarians can hardly expect to avoid just criticism for practising the methods of quackery under the thin guise of a high sounding titled medicine company. Every association should be jealous of the honorable and professional conduct of its members, and all members should be accorded like privileges.

Portland, Oregon, has added to her industries by establishing a horse-meat factory, where horseflesh is prepared for foreign markets. We firmly believe that these movements will ultimately sccure a place for horseflesh among our own people.

"The Humane and Economic Care of the Horse" was the topic presented in a recent address to the Humane Society of Kansas City, by Dr. J. H. Wattles, of the Kansas City Veterinary College.

Prof. R. S. Huidekoper has been selected as Veterinarian to the National Cat Show in New York in May.

